# Antibiotic Resistance Genes in Antibiotic-Free Chicken Farms

**DOI:** 10.3390/antibiotics9030120

**Published:** 2020-03-13

**Authors:** Yuhong Liu, Michael Dyall-Smith, Marc Marenda, Hang-Wei Hu, Glenn Browning, Helen Billman-Jacobe

**Affiliations:** 1 Asia-Pacific Centre for Animal Health, Department of Veterinary Biosciences, Faculty of Veterinary and Agricultural Sciences, Melbourne Veterinary School, The University of Melbourne, Parkville, VIC 3010, Australia; yuhongl@student.unimelb.edu.au (Y.L.); mike.dyallsmith@gmail.com (M.D.-S.); mmarenda@unimelb.edu.au (M.M.); glenfb@unimelb.edu.au (G.B.); 2 National Centre for Antimicrobial Stewardship, The Peter Doherty Institute, Elizabeth St Melbourne, VIC 3000, Australia; 3 School of Agriculture and Food, Faculty of Veterinary and Agricultural Sciences, The University of Melbourne, Parkville, VIC 3010, Australia; hang-wei.hu@unimelb.edu.au

**Keywords:** poultry, chicken, manure, microbiota, resistome

## Abstract

Rising concern about the use of antibiotics in food production has resulted in many studies on the occurrence of antibiotic resistance genes (ARGs) in animal-associated bacterial communities. There are few baseline data on the abundance of ARGs on farms where chickens are intensively raised with little or no use of antibiotics. This study used a high-throughput quantitative PCR array to survey two antibiotic-free chicken farms for the occurrence of ARGs and mobile genetic elements known to enhance the spread of ARGs. No antibiotics had been used on the study farms for five years prior to this study. The results provide a baseline for the occurrence of resistance genes in the chicken production system without direct selective pressure.

## 1. Introduction

Antibiotic usage in food animals raises concerns over the potential emergence and spread of resistant bacteria [1,2]. To limit the negative impacts, many countries have restricted or banned the use of specific antibiotics in food animals, especially those antibiotics used for nontherapeutic purposes or that are of high importance for human use [2]. In Australia, fluoroquinolones have never been approved for use in food animals, and the use of third generation cephalosporins is restricted [3]. In poultry, chlortetracycline is registered for the treatment of egg-producing chickens (layers), and several antibiotics, including amoxycillin, neomycin, lincomycin, spectinomycin and oxytetracycline, are registered for use in meat-producing chickens (broilers) [4]. Virginiamycin and zinc-bacitracin are allowed for the prevention and treatment of necrotic enteritis [4].

Previous studies of specific bacterial species isolated from Australian poultry have revealed antibiotic resistance. Some *Escherichia coli* isolates from chickens were resistant to tetracycline, ampicillin, trimethoprim-sufamethoxazole, streptomycin, spectinomycin, neomycin and florfenicol [5]. *Enterococcus* isolates have been reported with resistance to lincomycin, bacitracin, tetracycline and tylosin but no resistance to vancomycin or virginiamycin was reported [6]. *Campylobacter* isolates were found to be resistant to ampicillin, tetracycline and lincomycin, but no resistance to ciprofloxacin, gentamicin, erythromycin and tylosin was reported [4,7].

Sampling of individual animals is not always practical, especially when animals are raised on a large scale, such as caged chickens in commercial farms. In this case, environmental sampling is a cost-effective and efficient method to collect biomass representing a large population, and this has been used for monitoring the prevalence of pathogens in chicken flocks [8,9]. High-throughput quantitative PCR (HT-qPCR) and deep sequencing approaches have been used in a variety of human, animal and environmental samples to characterize the diversity of antibiotic resistance genes (ARGs) [10,11,12,13,14]. These methods are efficient and effective for the broad-spectrum detection and quantification of ARGs in complex samples.

The aim of this study was to assess the presence and diversity of ARGs and mobile genetic elements (MGEs) in poultry farms that had not used antibiotics for 5 years. The WaferGen HT-qPCR system was used to detect and quantify ARGs and MGEs in environmental samples from two chicken farms. The PCR primers targeted ARGs of the major antibiotic classes, and integrases and transposase genes. Caged chicken sheds from two types of farms were selected for investigation; the first was an egg production enterprise which housed caged layer chickens (Farm L), while the second was a broiler breeder farm (Farm B). The results should provide useful baseline information regarding ARG prevalence in the chicken production system without direct selective pressure.

## 2. Results

### 2.1. ARGs and MGEs in the Caged Layer Shed in Farm L

#### 2.1.1. Number of ARGs and MGEs 

**Figure 1:**
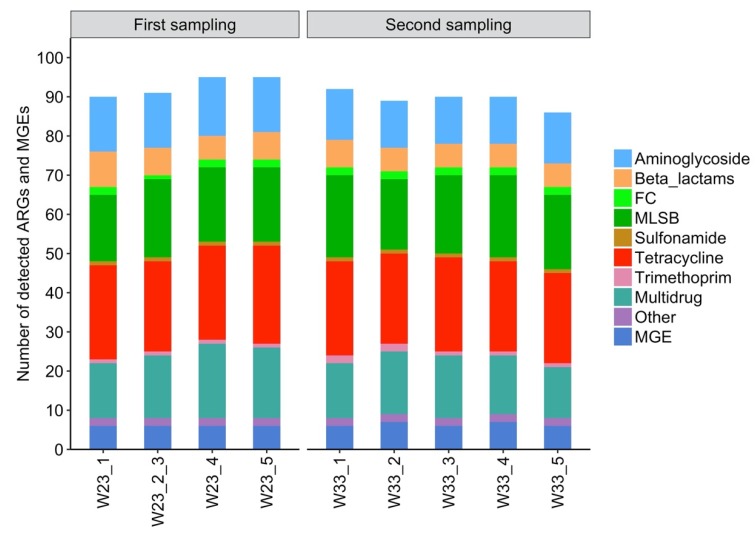
Number of detected antibiotic resistance genes (ARGs) and mobile genetic elements (MGEs) in each manure belt swab from the caged layer shed of each sampling visit on Farm L. The sampling was performed when the chickens were 23 weeks (W23) and 33 weeks old (W33). Sample 2 and 3 for W23 were combined to provide a sufficient biomass for processing. FC, fluoroquinolone/florfenicol/chloramphenicol; MLSB, macrolide-lincosamide-streptogramin B; MGE, mobile genetic element.

The caged layer shed on farm L was sampled twice when the chickens were 23 and 33 weeks old. Five manure belt swabs were collected from the shed each time. The biomass from two manure belt swabs in the first sampling were combined (sample W23_2 and W23_ 3) in order to have sufficient biomass for DNA extraction. The ARGs and MGEs in the DNA extracts were quantified using the WaferGen HT-qPCR system and the detection limit was set at Ct 27. Most of the ARGs were below the limits of detection. The combined number of ARGs and MGEs detected in each sample were between 80 and 89, out of the 285 ARGs tested, and either 6 or 7 out of 10 MGEs tested (Figure 1). The proportions of the classes of ARGs did not change substantially between samples.

#### 2.1.2. Abundance of ARGs and MGEs 

**Figure 2:**
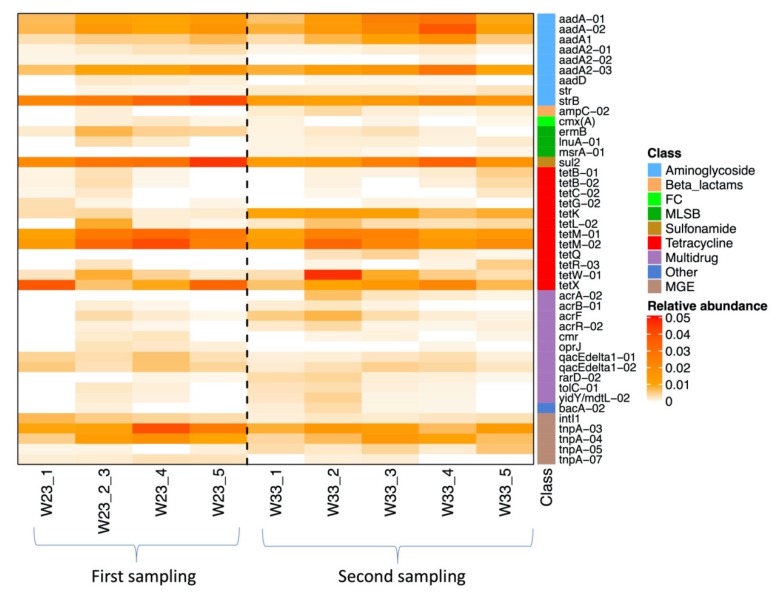
Heatmap of the relative abundance of ARGs and MGEs in each manure swab sample in Farm L. Only genes with a relative abundance above 10^−3^ in at least four samples are shown. FC, fluoroquinolone/florfenicol/chloramphenicol; MLSB, macrolide-lincosamide-streptogramin B; MGE, mobile genetic element.

The relative abundance of ARGs within their bacterial community was represented by their proportion relative to the 16S rRNA gene for each individual sample (Figure 2). The Ct for the 16S rRNA gene ranged between 8 and 12 and, after normalisation, the lowest relative abundance of ARG or MGE genes ranged between 10^−6^ and 10^−5^. ARGs with relative abundances above 10^−3^ in all the nine manure belt swab samples were aminoglycoside resistance genes *aad*A, *aad*A1, *aad*A2 and *str*B, sulfonamide resistance gene *sul*2 and tetracycline resistance genes *tet*M, *tet*K and *tet*X (Table 1). The *cph*A gene was detected at high abundance (~ 10^−1^) in one manure belt swab from the first sampling, but the gene was below the detection limit in the next sampling.

#### 2.1.3. Microbiota Analysis of Manure Belt swabs of Farm L

A total of 316,232 reads of the four manure belt swabs from the first sampling passed the quality filter. The original read depth of all samples ranged between 68,312 and 86,951. Reads of each sample were rarefied to 68,312, which was sufficient for the accurate analysis of microbiota richness and evenness. The rarefied reads were partitioned into 450 ASVs and assigned with taxonomic information.

**Figure 3:**
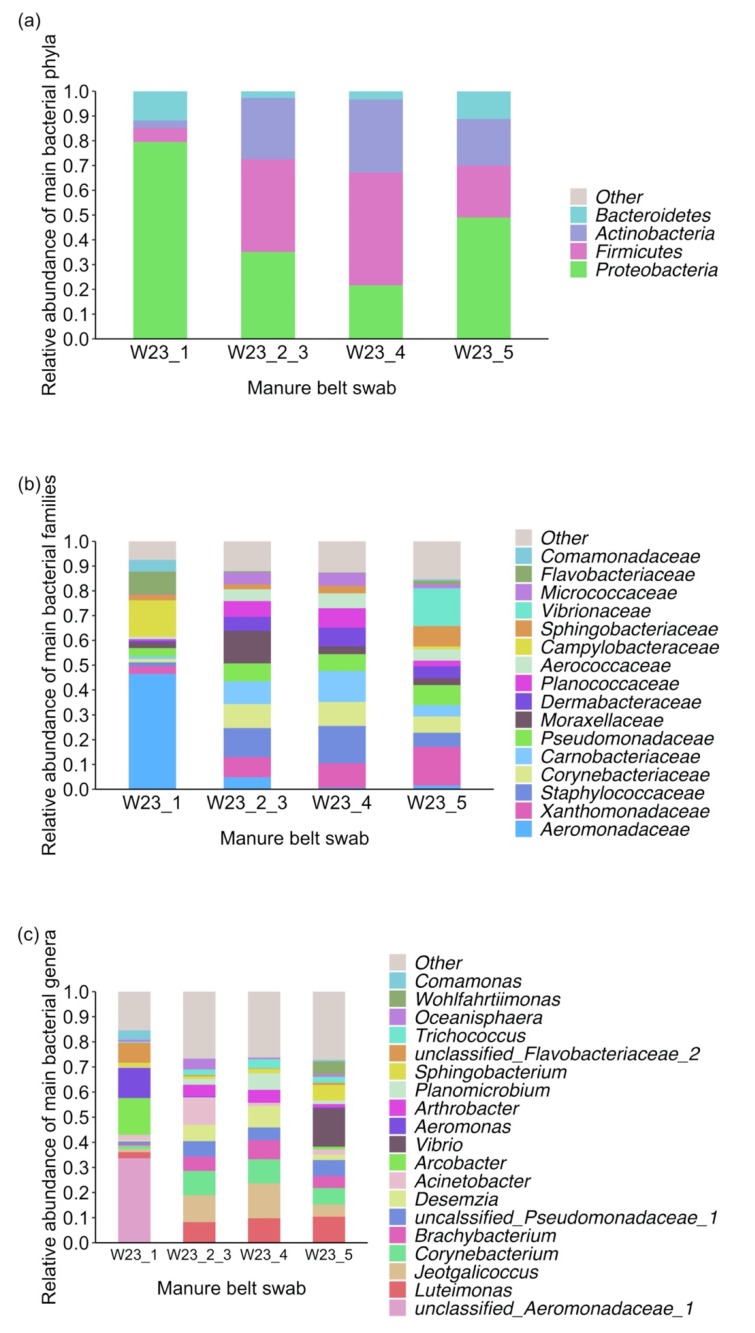
Relative abundance of dominant bacterial phyla (**a**), families (**b**) and genera (**c**) in the manure belt swabs of the first sampling in Farm L. Only taxa with relative abundance above 3% in at least one sample are shown. Taxa below the cut off were assigned as “other”.

*Proteobacteria* was the most dominant phylum in sample W23_1 and W23_5, which accounted for 80% and 49% of the total reads, respectively (Figure 3a). In sample W23_2_3 and W23_4, the most abundant phylum was *Firmicutes*, which accounted for 37% and 45% of the total reads, respectively (Figure 3a). *Actinobacteria* was the next most abundant phylum in sample W23_2_3, W23_4 and W23_5, which accounted for 25%, 30% and 19% of the total reads, respectively (Figure 3a). 

*Proteobacteria* in sample W23_1 was mainly represented by bacteria belonging to the *Aeromonadaceae* family and *Arcobacter* genus (*Campylobacteraceae* family), which accounted for 46% and 15% of the total reads, respectively (Figure 3c). In the other three samples, genus *Luteimonas*, *Jeotgalicoccus*, *Corynebacterium* and *Brachybacterium* were predominant in the microbiota, which together represented 27% to 41% of the total reads (Figure 3c). Moreover, sample W23_2_3 had a high level of *Acinetobacter* genus (11%) and sample W23_5 had a high level of *Vibrio* genus (15%) (Figure 3c). 

### 2.2. ARGs and MGEs in the Caged Broiler Breeder Sheds in Farm B

#### 2.2.1. Number and Abundance of ARGs and MGEs

The results of Farm L showed that the Ct of many of ARGs was above the cutoff of 27. It was not cost-effective to include all of the 295 ARGs and MGEs in further testing. Consequently, the HT-qPCR assay was reduced to 45 ARGs and four MGEs for samples from Farm B. 

Three sheds of caged broiler breeder chickens were sampled on Farm B and 24 manure belt swabs were collected from each shed. Samples that failed to amplify the 16S rRNA gene were excluded in the analysis, leaving 22 swabs that were validated for sheds A and B, respectively, and 16 swabs in shed C. 

**Figure 4:**
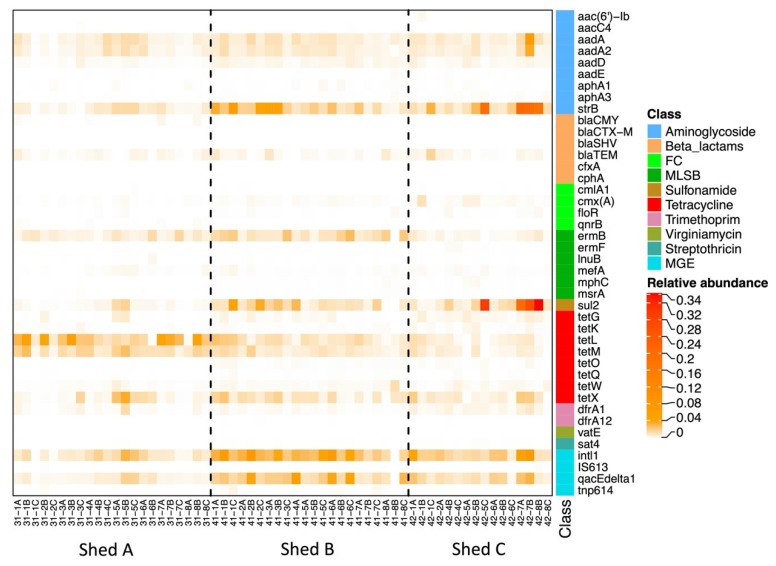
Heatmap of the relative abundance of ARGs and MGEs in all manure belt samples from three sheds in Farm B. FC, fluoroquinolone/florfenicol/chloramphenicol; MLSB, macrolide-lincosamide-streptogramin B; MGE, mobile genetic element.

Each shed was detected with 44 genes and the three sheds shared 42 genes. The relative abundance of genes detected in Farm B is shown in the heatmap (Figure 4). A total of 34 genes were detected in more than 80% samples (48 out of 60). The most abundant ARG was the aminoglycoside resistance gene *str*B, with a median relative abundance of 1.3 × 10^−2^. The next most abundant ARG was tetracycline resistance gene *tet*L, with the median relative abundance of 1.2 × 10^−2^, and the other two abundant *tet* genes were *tet*M and *tet*X. Other abundant genes were *sul*2, *erm*B, *aad*A and *aad*A2. In addition, the class I integron markers *intI*1 and *qac*E∆ were detected at high levels in the sheds, with median relative abundances of 1.8 × 10^−2^ and 1.1 × 10^−2^, respectively (Table 2). Relative abundances of these genes were further compared among the sheds using a pairwise Wilcoxon rank sum test. Three genes (*tet*X, *aad*A and *aad*A2) showed no significant differences in abundance between sheds. Shed A had significantly different abundances of gene *tet*L, *tet*M, *str*B, *sul*2, *erm*B, *int*I1, *qac*E∆ compared to shed B and/or shed C (*p* < 0.05). Sheds B and C had significantly different abundances of gene *tet*L, *erm*B, i*nt*I1 and *qac*E∆ (*p* < 0.05). The ARGs with high human clinical importance, such as the beta lactamase resistance genes *bla*SHV, *bla*CTX-M, *cph*A, the fluoroquinolone resistance gene *qnr*B and the virginiamycin resistance gene *vat*E, detected at very low abundances (~10^−6^ to 10^−5^) in the samples.

#### 2.2.2. Similarity of ARG and MGE Profiles between Sheds and Farms

**Figure 5:**
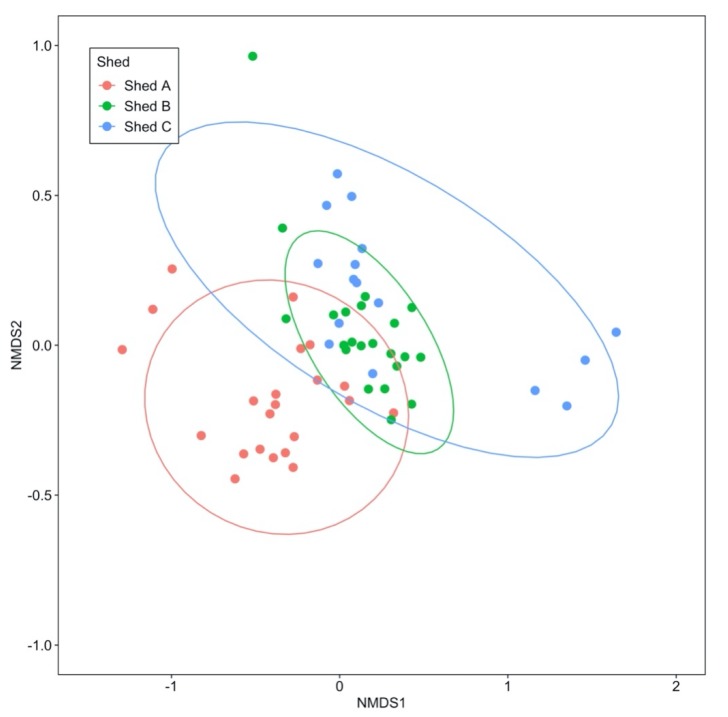
NMDS using Bray–Curtis distances to compare the similarity of ARG profiles of the manure belt swabs between the sheds in the broiler breeder Farm B. The ellipses indicate 95% confidence region of each cluster.

NMDS analysis using Bray–Curtis distance derived from the relative abundance of ARGs and MGEs was used to explore sample clustering based on sheds in Farm B. The results are shown in Figure 5 and reveal a separation of shed A from sheds B and C (R = 0.39, *p* = 0.001, ANOSIM).

**Figure 6:**
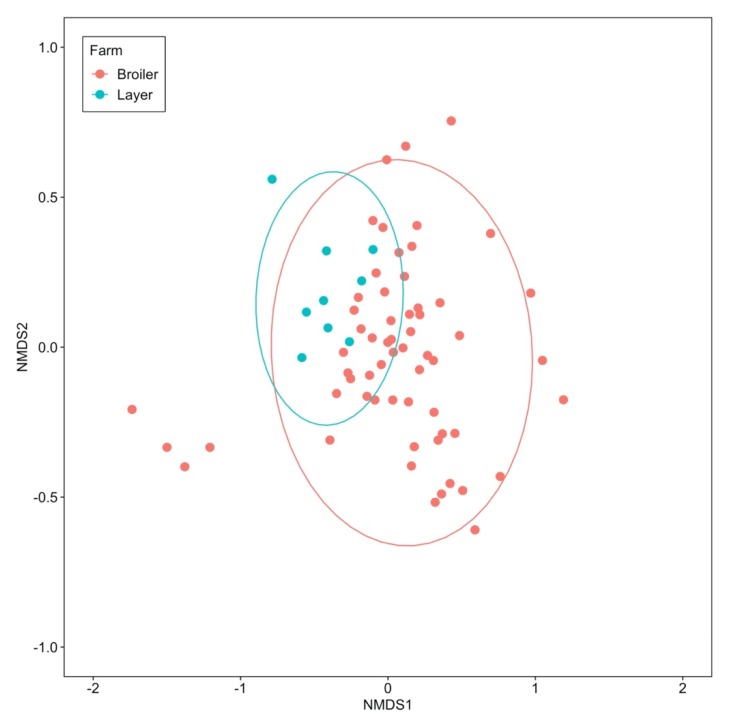
NMDS using Bray–Curtis distances to compare the similarity of the manure belt swab ARG profiles between layer Farm L and broiler breeder Farm B. The analysis was based on the relative abundance of 33 ARGs detected in both farms. The ellipses indicate 95% confidence region of each cluster.

The ARG profile of manure belt swabs of Farm B was further compared to that of the layer Farm L. NMDS analysis was based on the relative abundance of 33 ARGs detected in both farms. No significant difference was found between the two farms (R = 0.17, *p* = 0.08, ANOSIM) (Figure 6).

## 3. Discussion

This work explored the ARG profile in two antibiotic-free farms, a layer farm and a broiler breeder farm. Manure belt swabs were used for sampling as this was easy to perform in a large chicken shed, and the biomass collected on the swabs represented a large number of birds. The manure belt swabs collected in layer Farm L showed a high abundance of *tet*M, *sul*2 and *str*B, which agreed with the results of previous studies that examined the resistome of intensively reared chickens [15,16]. Indeed, tetracycline resistance genes, particularly, *tet*Q, *tet*W and *tet*M, have been found to be ubiquitous in faecal specimens from human and animals [11,14,17]. 

The *sul*2 gene is plasmid borne, and has been found in plasmids from different incompatibility groups and often linked to streptomycin resistance genes *str*A and *str*B [18,19,20]. A recent study reported that the *sul*2-*str*A-*str*B cluster could be found in ice core samples from Antarctica that were over 1000 years old, suggesting that the formation and dissemination of this ARG cluster can happen without human interventions involving antibiotic use [21]. In the current study, no antibiotics had been used on this farm for more than 5 years, so the prevalence of these ARGs in this layer farm was not likely driven by antibiotic selective pressure and is more likely a reflection of their baseline abundances in the farming environment.

Analysis of the microbiota profile of the manure belt swabs showed a dominance of family *Aeromonadaceae* and genus *Luteimonas*, *Jeotgalicoccus*, *Corynebacterium* and *Brachybacterium* in the samples. The *Corynebacterium* genus was found abundant in chicken duodenum, jejunum, ileum, and colon [22]. However, other genera, such as *Lactobacillus*, *Enterococcus*, *Bacteriodes* and *Ruminococcus,* reported as abundant in the chicken gastrointestinal tract [22,23], were rarely detected in our results. The divergency might be explained by the differences between samples. Previous studies usually collected fresh chicken faeces or gut contents, while, in our study, faecal material collected from the manure belt would have been exposed and the faecal microbiota could be impacted by environmental factors, such as oxygen, temperature and moisture. Indeed, *Luteimonas, Corynebacterium* and *Brachybacterium* were usually detected in chicken litter and *Corynebacterium* was one of the major genera in the litter microbiota [24,25].

The results of the Farm L study showed that many of the qPCRs were negative, which was consistent with the antibiotic-free policy of this farm. The results from the study of Farm L informed the experimental design of the study of Farm B, which also did not use antibiotics. A reduced set of HT-qPCR primers was selected for use in the Farm B study, which allowed more samples to be analysed while still maintaining broad coverage of the most frequent ARGs and MGEs present.

Farm B samples had abundant tetracycline resistance genes in most samples. This is consistent with findings on Farm L and other studies quantifying ARGs in faecal samples from poultry [26,27], swine [14,28] and humans [11,17]. These *tet* genes are known to be present in a wide range of bacterial species [29], some of which are members of the dominant flora of the normal chicken gastrointestinal tract and chicken litter, such as *Clostridium*, *Lactobacillus, Bacteroides* and *Corynebacterium* [23]. Metagenomic studies have shown that the ribosome protection type *tet* genes contribute about 30% to the total ARG abundance in the chicken cecum [30], and many *tet* genes are often associated with mobile elements in diverse species [31,32,33,34,35]. The macrolide resistance gene *erm*B was also found to be abundant in samples across all broiler sheds. Similar to the *tet* genes, *erm*B was also detected in a wide range of bacterial species [29] and associated with conjugative transposons [36,37,38], which may explain the wide dissemination of the gene in the environment.

Moreover, the ARG profile of the manure belt swabs from Farm B was similar to that from the Farm L. The comparison included ARGs that were detected at high levels in both farms, such as *tet*M, *tet*X, *str*B and *sul*2. As both farms were antibiotic-free for at least 5 years prior to sampling, the result indicates that those ARGs were ubiquitous in poultry manure and their presence was not necessarily related to human antibiotic usage.

In conclusion, this study showed that HT-qPCR can be used for surveying chicken farm environments, and that manure belt sampling is both convenient and useful as long as the physical design of the housing permits access to the belt. The ARG profiles for the two farms were similar. The ARGs with higher abundances, particularly *tet*M, *str*B and *sul*2, were likely due to their carriage by bacterial species naturally present in the chicken faecal microbiota, or because these genes have been already spread widely in the environment and were not the result of selection due to antibiotic use.

## 4. Materials and Methods 

### 4.1. Study Farms and Chickens 

In the layer Farm L, birds in the caged shed were kept in cages with six birds per cage. The study shed housed around 65,000 birds in five frames six tiers high. Manure accumulated on a conveyor belt beneath each tier of cages and was removed weekly. 

In the broiler breeder Farm B, the birds were housed in frames three to four tiers high. Each shed contained eight frames which housed around 24,000 birds in total. Three sheds on the farm were sampled. Birds in shed B and C were originally from the same cohort of chicks and birds in shed A were from a different cohort. 

### 4.2. Sampling 

The caged layer shed in Farm L was sampled twice when the birds were 23 and 33 weeks old, respectively. One manure belt per frame was sampled and there were five frames in total. In Farm B, three manure belts of each frame were sampled, and 24 swabs were collected from each shed.

The manure belts were sampled according to the method described in [9]. Manure belt swabs were collected by wiping the edge of one end of the manure belt, using sterile 10 × 10cm cotton gauze swabs (ZebraVet, Australia) premoistened with buffered peptone water. Swabs were stored in sterile plastic bags and transported to the laboratory at ambient temperature. Samples from Farm L were transported to the laboratory on the same day of collection, and samples from Farm B were transported to the laboratory the next morning within 24h of collection. Upon receipt, the biomass was collected from the swabs and frozen at -80°C until DNA extraction. 

### 4.3. DNA Extraction 

The swabs were rinsed in 50ml peptone and the biomass was collected by centrifugation at 2885× *g* for 30min at 4 °C (Allegra X-12R centrifuge, Beckman Coulter, USA). The pellet was stored in one 2ml microcentrifuge tube and stored at −80 °C until DNA extraction. 

DNA was extracted using the DNeasy PowerSoil Kit (Qiagen, Carlsbad, CA, USA) according to the manufacturer’s instructions, except that the biomass mixed with buffer C1 was processed with a FastPrep homogeniser (Bio101, USA) at 5.5m/s for 30s. DNA quality and quantity were checked by Nanodrop (Thermo Fisher Scientific, USA) and Qubit (Thermo Fisher Scientific, USA), respectively. DNA was store at −20 °C until analysis. 

### 4.4. HT-qPCR Array for Detection and Quantification of ARGs and MGEs

ARGs and MGEs were detected by the WaferGen SmartChip Real-time PCR system (WaferGen Inc. USA).

For Farm L, the array contained 296 validated primers targeting 285 ARGs, 10 mobile genetic elements (MGEs), and one 16S rRNA gene (Table S1) [13,14]. The assay includes genes from all major classes of ARGs, including aminoglycoside, beta lactamase, fluoroquinolone/florfenicol/chloramphenicol (FC), macrolide-lincosamide-streptogramin B (MLSB), sulfonamide, trimethoprim, tetracycline, vancomycin, multidrug resistance and other resistance genes. For Farm B, 45 primers were used to amplify ARGs, four primer pairs were used to amplify MGEs and one primer pair was specific to the bacterial 16S rRNA gene (Table S2). The qPCR conditions have been described previously [39]. The cycle threshold (Ct) cut-off was 27 [14]. The abundance of each ARG was normalised to the 16S rRNA gene in each sample using the equation Relative abundance =$\;\frac{E{\left(16S\right)}^{\mathrm{Ct}\left(16S\right)}}{E{\left(\mathrm{ARG}\right)}^{\mathrm{Ct}\left(\mathrm{ARG}\right)}}$, where E(16S) is the efficiency of the 16S rRNA gene, E(ARG) is the efficiency of each ARG or MGE, and Ct is the threshold cycle [40,41].

### 4.5. Bacterial 16S rRNA Sequencing and Bioinformatic Analyses 

Bacterial communities in the manure belt swabs (Farm L) of the first sampling were characterised by sequencing the V3-V4 region of the 16S rRNA gene using the Illumina MiSeq platform. Primers were as follows: forward primer (342F): 5′-CCTAYGGGRBGCASCAG-3′, reverse primer (806R): 5′-GGACTACNNGGGTATCTAA-3′ [42,43]. Sequences were processed through the QIIME2 pipeline (Version qiime2-2018.11) [44]. De-multiplexed reads were trimmed to 250bp, de-noised, paired and grouped into amplicon sequence variants (ASVs) by DADA2 [45]. ASVs only present in a single sample or classified as non-bacteria were removed. Taxonomy was assigned using a pre-trained Naïve Bayes classifier with the Greengenes database (2013 August release) [46,47]. For fair comparison, sequence depth was equalised by randomly subsampling the same number of reads of each sample from the original dataset. The minimum number of reads that would not affect coverage or discard any samples was used.

### 4.6. Statistical Analysis 

A heatmap of the relative abundance of each gene in each sample of Farm L and Farm B was generated using the ComplexHeatmap R package [48]. 

Differences in the relative abundance of ARGs between sheds in Farm B were tested by pairwise Wilcoxon rank sum test with P value adjusted by the Benjamini–Hochberg correction method [49] using the basic stats R package. A P value less than 0.05 was considered as a significant difference.

The similarity of ARG profiles of the manure belt swabs between sheds in Farm B was compared using the NMDS method with Bray–Curtis distance matrix based on the relative abundance of ARGs and MGEs. Similarity between sheds was tested by analysis of similarity (ANOSIM) using the ‘anosim’ function in the vegan package of R. NMDS analysis results were plotted using the ggplot2 R package with ellipses indicating the 95% confidence region of each cluster.

The similarity of ARG profiles of manure belt swabs between Farm L and Farm B was tested based on the relative abundance of ARGs tested in both farms, using the NMDS and ANOSIM methods described above.

## Figures and Tables

**Table 1:** Most abundant ARGs in the layer Farm L.

Gene	Relative Abundance		
	Min	Max	Median
*aad*A	6.5 × 10^−3^	2.9 × 10^−2^	1.2 × 10^−2^
*aad*A1	3.4 × 10^−3^	1.9 × 10^−2^	6.0 × 10^−3^
*aad*A2	1.0 × 10^−3^	3.7 × 10^−3^	2.1 × 10^−3^
*str*B	1.2 × 10^−2^	4.0 × 10^−2^	2.3 × 10^−2^
*sul*2	1.3 × 10^−2^	4.5 × 10^−2^	2.5 × 10^−2^
*tet*K	1.9 × 10^−3^	1.3 × 10^−2^	7.5 × 10^−3^
*tet*M	1.1 × 10^−2^	3.2 × 10^−2^	2.3 × 10^−2^
*tet*W	2.4 × 10^−3^	4.6 × 10^−2^	4.9 × 10^−3^
*tet*X	6.7 × 10^−3^	3.7 × 10^−2^	1.1 × 10^−2^

**Table 2:** Most abundant ARGs and MGEs in the broiler breeder Farm B.

Gene	Relative Abundance		
	Min	Max	Median
*aad*A	6.4 × 10^−4^	6.7 × 10^−2^	7.2 × 10^−3^
*aad*A2	5.0 × 10^−4^	3.8 × 10^−2^	5.6 × 10^−3^
*str*B	7.0 × 10^−4^	2.1 × 10^−1^	1.3 × 10^−2^
*erm*B	1.4 × 10^−3^	3.0 × 10^−2^	6.3 × 10^−3^
*sul*2	1.5 × 10^−4^	3.4 × 10^−1^	8.0 × 10^−3^
*intl*1	6.8 × 10^−4^	7.9 × 10^−2^	1.8 × 10^−2^
*qac*E∆	1.3 × 10^−3^	5.4 × 10^−2^	1.1 × 10^−2^
*tet*L	1.0 × 10^−3^	5.5 × 10^−2^	1.2 × 10^−2^
*tet*M	1.8 × 10^−3^	3.0 × 10^−2^	9.2 × 10^−3^
*tet*X	2.2 × 10^−4^	3.8 × 10^−2^	7.7 × 10^−3^

## Supplementary Materials

The following are available online at https://www.mdpi.com/2079-6382/9/3/120/s1, Table S1: qPCR primers for ARGs and MGEs in Farm L, Table S2: qPCR primers for ARGs and MGEs in Farm B.
